# Comparative Genomics of a Parthenogenesis-Inducing *Wolbachia* Symbiont

**DOI:** 10.1534/g3.116.028449

**Published:** 2016-05-16

**Authors:** Amelia R. I. Lindsey, John H. Werren, Stephen Richards, Richard Stouthamer

**Affiliations:** * Department of Entomology, University of California, Riverside, California, 92521; † Department of Biology, University of Rochester, New York, 14627; ‡ Human Genome Sequencing Center, Department of Human and Molecular Genetics, Baylor College of Medicine, Houston, Texas, 77030

**Keywords:** *Trichogramma*, gene truncations, symbiosis, genome content, Rickettsiales

## Abstract

*Wolbachia* is an intracellular symbiont of invertebrates responsible for inducing a wide variety of phenotypes in its host. These host-*Wolbachia* relationships span the continuum from reproductive parasitism to obligate mutualism, and provide a unique system to study genomic changes associated with the evolution of symbiosis. We present the genome sequence from a parthenogenesis-inducing *Wolbachia* strain (*w*Tpre) infecting the minute parasitoid wasp *Trichogramma pretiosum*. The *w*Tpre genome is the most complete parthenogenesis-inducing *Wolbachia* genome available to date. We used comparative genomics across 16 *Wolbachia* strains, representing five supergroups, to identify a core *Wolbachia* genome of 496 sets of orthologous genes. Only 14 of these sets are unique to *Wolbachia* when compared to other bacteria from the Rickettsiales. We show that the B supergroup of *Wolbachia*, of which *w*Tpre is a member, contains a significantly higher number of ankyrin repeat-containing genes than other supergroups. In the *w*Tpre genome, there is evidence for truncation of the protein coding sequences in 20% of ORFs, mostly as a result of frameshift mutations. The *w*Tpre strain represents a conversion from cytoplasmic incompatibility to a parthenogenesis-inducing lifestyle, and is required for reproduction in the *Trichogramma* host it infects. We hypothesize that the large number of coding frame truncations has accompanied the change in reproductive mode of the *w*Tpre strain.

*Wolbachia* is a maternally transmitted, intracellular symbiont of arthropods and nematodes that exhibits a range of complex interactions with its hosts (Werren 1997; Werren *et al.* 2008; Stouthamer *et al.* 1999a). It is estimated to infect 40–60% of arthropod species (Zug and Hammerstein 2012; Hilgenboecker *et al.* 2008). Across the arthropods, *Wolbachia* is well known for modifying host reproduction, by utilizing various mechanisms that enhance fitness or numbers of infected females. By promoting infected females, *Wolbachia* ensures its own maternal transmission and has the ability to spread rapidly through a population (Walker *et al.* 2011; Weeks *et al.* 2007; Turelli and Hoffmann 1991). These reproductive modifications include: cytoplasmic incompatibility (CI), male killing, feminization, and parthenogenesis-induction (PI) (Werren 1997). In addition to these reproductive phenotypes, some *Wolbachia* strains protect against pathogens (Chrostek *et al.* 2013; Moreira *et al.* 2009; Kambris *et al.* 2010), supply essential nutrients to their hosts (Nikoh *et al.* 2014; Hosokawa *et al.* 2010), are required for successful egg development (Kremer *et al.* 2009; Timmermans and Ellers 2009; Dedeine *et al.* 2001), or are essential for the production of female offspring (Russell and Stouthamer 2011; Stouthamer *et al.* 2010). In filarial nematodes, *Wolbachia* is an obligate mutualist providing a diversity of benefits to its host, including evasion of the vertebrate immune system (Darby *et al.* 2012). For these reasons, *Wolbachia* has captured considerable interest in applied fields as a potential “agent” to modify pest populations, reduce pathogen loads in vectors, and specifically target filarial nematodes by way of their obligate symbionts (Zabalou *et al.* 2004; Bourtzis *et al.* 2014; Taylor *et al.* 2000).

In addition to the practical applications of studying *Wolbachia*, the complexity of interactions with diverse hosts provides an opportunity to explore genomic changes accompanying the evolution of such unique life histories. Nested within a clade of other symbiotic and pathogenic bacteria, *Wolbachia* are members of the Rickettsiales, an order of α-proteobacteria (O’Neill *et al.* 1992; Dumler *et al.* 2001). The *Wolbachia* clade is composed of 16 reported supergroups, denoted A–F and H–Q (Ros *et al.* 2009; Augustinos *et al.* 2011; Bing *et al.* 2014; Haegeman *et al.* 2009; Lo *et al.* 2002; Glowska *et al.* 2015), with supergroups A–D being the most well studied. Supergroup G is no longer considered a distinct *Wolbachia* lineage, as it represents a recombinant between supergroups A and B (Baldo and Werren 2007). Supergroups A and B are a monophyletic assemblage infecting arthropods (Gerth *et al.* 2014), whereas supergroups C and D are the major nematode-infecting lineages (Bandi *et al.* 1998). Supergroup F is unique as it contains both nematode and arthropod-infecting strains (Casiraghi *et al.* 2005), including the bed bug-infecting *Wolbachia* strain *w*Cle that supplements B vitamins to its obligate blood-feeding hosts (Nikoh *et al.* 2014; Hosokawa *et al.* 2010). The less studied supergroups H–Q infect a variety of hosts, including termites, aphids, whiteflies, mites, fleas, and a plant-parasitic nematode (Ros *et al.* 2009; Augustinos *et al.* 2011; Bing *et al.* 2014; Haegeman *et al.* 2009; Lo *et al.* 2002; Glowska *et al.* 2015).

While cocladogenesis of *Wolbachia* and their hosts does occur (Raychoudhury *et al.* 2009), it is relatively uncommon, and host-switching is a prominent feature of *Wolbachia*’s evolutionary history (Vavre *et al.* 1999; van Meer *et al.* 1999; Zhou *et al.* 1998; Baldo *et al.* 2006). In addition to the incongruence of host and symbiont phylogenies, there is little conservation of the induced phenotypes. For example, independently derived parthenogenesis-inducing (PI) *Wolbachia* are found in the A and B supergroups (Stouthamer *et al.* 1993), and likely the F supergroup (Baldo *et al.* 2007). These PI-*Wolbachia* strains induce parthenogenesis through different mechanisms including the merging of nuclei (Gottlieb *et al.* 2002), a failed anaphase during the first embryonic cell division (Stouthamer and Kazmer 1994; Pannebakker *et al.* 2004), and functional apomixis (Weeks and Breeuwer 2001). Uninfected parasitoid wasps of the genus *Trichogramma* are arrhenotokous, but infection with PI-*Wolbachia* strains causes gamete duplication in unfertilized eggs by preventing chromosome segregation during anaphase of the first mitotic division of the egg, resulting in a diploid female (Stouthamer and Kazmer 1994). The PI-*Wolbachia* strains infecting *Trichogramma* spp. are unique for at least three reasons: there is a single origin of *Wolbachia* infection for the genus (Werren *et al.* 1995; van Meer *et al.* 1999); the *Trichogramma* hosts can evolve dependencies upon their *Wolbachia* infection for the production of females (Russell and Stouthamer 2011; Stouthamer *et al.* 2010); and, unlike other arthropod-infecting strains, the PI-*Wolbachia* infecting *Trichogramma* do not have relationships with phages (Gavotte *et al.* 2007).

*Wolbachia* genomes are small in size, ranging from 0.9–1.5 Mbp, and contain a number of unique features. The arthropod infecting genomes have a large number of repetitive and mobile elements, including ankyrin repeat domain-containing (ANK) genes (Iturbe-Ormaetxe *et al.* 2005; Siozios *et al.* 2013b; Papafotiou *et al.* 2011), bacteriophage sequences (Gavotte *et al.* 2007), transposons, and many copies of short open reading frames (ORFs) of unknown function (Wu *et al.* 2004). Little is known about the role that these short, unannotated ORFs play in the biology of *Wolbachia*.

Here, we explore the changes in genome content across *Wolbachia*, and present a draft genome for the PI-*Wolbachia* strain, *w*Tpre, infecting the parasitoid wasp *Trichogramma pretiosum*. The *w*Tpre genome represents the most complete PI-*Wolbachia* genome assembly to date, and the first B supergroup PI-*Wolbachia* genome. We show evidence for protein sequence truncation in 20% of the *w*Tpre gene set, and hypothesize that these truncations are a feature of the change in reproductive phenotype.

## Materials and Methods

### Biological materials

A unisexual colony of naturally *Wolbachia*-infected *T. pretiosum* was chosen for genome sequencing. Originally collected in the Puira Valley of Peru, this colony has been maintained in a commercial insectary since 1966 (Beneficial Insectary, Guelph, Ontario, Canada), and herein is referred to as the “Insectary Line.” Species identifications were confirmed by molecular protocols from Stouthamer *et al.* (1999b), and *Wolbachia* infection status was confirmed using the protocols from Stouthamer *et al.* (1990) and Werren and Windsor (2000). Attempts to initiate *Wolbachia*-free replicates of this colony following antibiotic treatment protocols from Stouthamer *et al.* (1990) have not been successful due to severe fertility reduction, as seen in Russell and Stouthamer (2011).

### Identification of a wTpre genome

The genome of the *T. pretiosum* Insectary Line (GenBank Accession Number: JARR00000000) (A. R. I. Lindsey *et al.*, unpublished results) was sequenced in collaboration with the i5k initiative to sequence 5000 arthropod genomes (www.arthropodgenomes.org/wiki/i5K) and made publicly available prior to publication under the Fort Lauderdale agreement. The *T. pretiosum* assembly was scanned for evidence of *Wolbachia* DNA using two methods. First, total DNA was extracted from 10 wasps using a Chelex method (Walsh *et al.* 1991) as implemented by Stouthamer *et al.* (1999b). The *Wolbachia* 16S rRNA gene was amplified and sequenced with W-Specf and W-Specr primers (Werren and Windsor 2000). Sequences were aligned and primer sequences excised in Sequencher 4.9. The 16S rRNA gene was then queried against the *T. pretiosum* genome assembly using nucleotide BLASTN at NCBI (http://blast.ncbi.nlm.nih.gov/Blast.cgi). The remaining scaffolds were checked for bacterial DNA sequences by querying them against Bacteria (taxid: 2) in NCBI GenBank with blastn. Second, the *T. pretiosum* assembly was scanned with the bioinformatics pipeline developed by Wheeler *et al.* (2013), in order to identify bacterial sequences from a eukaryotic background.

### Genome annotation, clusters of orthologous genes, and completeness estimates

The IGS Annotation Engine was used for structural and functional annotation of the *w*Tpre genome (http://ae.igs.umaryland.edu/cgi/index.cgi, Galens *et al.* 2011). Manatee was used to view annotations (http://manatee.sourceforge.net/). The *w*Tpre genome and 17 other previously published genomes (see Table 1) were used in comparative analyses. Previously published genomes were reannotated with the IGS Annotation Engine, and Clusters of Orthologous Genes (COGs) across all 18 genomes were defined using Sybil (http://sybil.sourceforge.net/index.html, Riley *et al.* 2012; Crabtree *et al.* 2007). Genome completeness was assessed with the BUSCO pipeline (Simão *et al.* 2015) using the 40 core bacterial genes from Mende *et al.* (2013) compared to the gene set from each *Wolbachia* genome (-m = OGS).

**Table 1:** *Wolbachia* strains used in comparative and phylogenetic analyses

Strain	Host	Supergroup	Size (bp)	ORFs	Reference	Accession Number	BUSCO Score*^a^*
*w*Gmm	*Glossina morsitans morsitans*	A	1,019,687	1378	Brelsfoard *et al.* (2014)	AWUH00000000	C: 77.5% [D: 6.4%], F: 5%, M: 17.5%, n: 40
*w*Ha	*Drosophila simulans*	A	1,295,804*^b^*	1342	Ellegaard *et al.* (2013)	CP003884	C: 85% [D: 2.9%], F: 5%, M: 10%, n: 40
*w*Mel	*Drosophila melanogaster*	A	1,267,782*^c^*	1401	Wu *et al.* (2004)	AE017196	C: 87.5% [D: 2.9%], F: 2.5%, M: 10%, n: 40
*w*Ri	*Drosophila simulans*	A	1,445,873*^c^*	1493	Klasson *et al.* (2009)	CP001391	C: 82.5% [D: 3%], F: 5%, M: 12.5%, n: 40
*w*Suzi	*Drosophila suzukii*	A	1,415,350	1528	Siozios *et al.* (2013a)	CAOU00000000	C: 87.5% [D: 2.9%], F: 2.5%, M: 10%, n: 40
*w*AIbB	*Aedes albopictus*	B	1,162,431	1187	Mavingui *et al.* (2012)	CAGB00000000	C: 82.5% [D: 3%], F: 2.5%, M: 15%, n: 40
*w*Bol1	*Hypolimnas bolina*	B	1,377,933	1369	Duplouy *et al.* (2013)	CAOH00000000	C: 80% [D: 3.1%], F: 5%, M: 15%, n: 40
*w*Di	*Diaphorina citri*	B	1,240,904	1250	Saha *et al.* (2012)	AMZJ00000000	C: 80% [D: 3.1%], F: 2.5%, M: 17.5%, n: 40
*w*No	*Drosophila simulans*	B	1,301,823*^c^*	1317	Ellegaard *et al.* (2013)	CP003883	C: 82.5% [D: 3%], F: 2.5%, M: 15%, n: 40
*w*Pip_Pel	*Culex quinquefasciatus* Pel	B	1,482,355*^c^*	1461	Klasson *et al.* (2008)	AM999887	C: 80% [D: 3.1%], F: 5%, M: 15%, n: 40
*w*Pip_JBH	*Culex quinquefasciatus* JBH	B	1,542,137	1556	Salzberg *et al.* (2009)	ABZA00000000	C: 75% [D: 3.3%], F: 2.5%, M: 22.5%, n: 40
*w*Pip_Mol	*Culex pipiens molestus*	B	1,340,443*^c^*	1340	Pinto *et al.* (2013)	HG428761	C: 80% [D: 3.1%], F: 2.5%, M: 17.5%, n: 40
*w*Tpre	*Trichogramma pretiosum*	B	1,133,709*^b^*	1405	This study	LKEQ00000000	C: 77.5% [D: 3.2%], F: 5%, M: 17.5%, n: 40
*w*VitB	*Nasonia vitripennis*	B	1,107,643	1245	Kent *et al.* (2011)	AERW00000000	C: 77.5% [D: 3.2%], F: 2.5%, M: 20%, n: 40
*w*Oo	*Onchocerca ochengi*	C	957,990*^c^*	1272	Darby *et al.* (2012)	HE660029	C: 75% [D: 3.3%], F: 2.5%, M: 22.5%, n: 40
*w*Bm	*Brugia malayi*	D	1,080,084*^c^*	1339	Foster *et al.* (2005)	AE017321	C: 82.5% [D: 3%], F: 5%, M: 12.5%, n: 40
*w*Wb	*Wuchereria bancrofti*	D	1,052,327	2144	Desjardins *et al.* (2013)	ADHD00000000	C: 45% [D: 0%], F: 20%, M: 35%, n: 40
*w*Cle	*Cimex lectularius*	F	1,250,060*^c^*	1357	Nikoh *et al.* (2014)	AP013028	C: 72.5% [D: 3.4%], F: 2.5%, M: 25%, n: 40

ORFs, open reading frames; BUSCO, benchmarking universal single-copy orthologs; C, complete; D, duplicated; F, fragmented; M, missing; n, number of genes used.

a BUSCO scores in standard BUSCO notation.

b Single-scaffold assembly.

c Complete assembly.

### Phylogenetic analyses

A phylogenetic reconstruction of *Wolbachia* strains was inferred using the five Multi Locus Sequence Typing (MLST) genes (Baldo *et al.* 2006), with *Anaplasma marginale* str. *Florida* (GenBank Accession Number: PRJNA58577) “Ama” as an outgroup. In addition to the strains in Table 1 (minus *w*Wb, see *Results*), we included *Wolbachia* strains from the MLST database (*w*Ajap infecting *Asobara japonica*, *w*Uni infecting *Muscidifurax uniraptor*, *w*Dali infecting *Diaphorencyrtus aligarhensis*, *w*Tdei infecting *Trichogramma deion*, *w*Efor infecting *Encarsia formosa*, *w*PsiaB infecting *Protocalliphora sialia*, and *w*Lcla infecting *Leptopilina clavipes*) and the *w*Tbras strain infecting *Trichogramma brassicae* (downloaded from GenBank, Accession Numbers: JF920468.1, JF920470.1, JF920472.1, JF920464.1, and JF920466.1). Multiple alignments were created for each gene using the L-INS-i algorithm in MAFFT version 7 (Katoh and Standley 2013), and were concatenated prior to maximum likelihood analyses in RAxML version 8.2.4 (Stamatakis 2014) using the GTRGAMMA substitution model and 1000 bootstrap replicates. A second phylogenetic reconstruction was made using the same methods, but with only the strains used in our comparative analyses. Trees were visualized in FigTree version 1.4.2 (http://tree.bio.ed.ac.uk/software/figtree/) and annotated in Inkscape (https://inkscape.org/en/).

### Identification of core and unique genomes

Unique and core genome assessments were performed using Sybil results loaded on a Chado relational database (Galens *et al.* 2011; Mungall *et al.* 2007). The core genome was determined by identifying all COGs that had at least one gene member from each *Wolbachia* strain being considered. COGs were considered unique to a monophyletic assemblage when all members of the COG belonged exclusively to the clade, and were found in all members of the clade. To determine the uniqueness of the *Wolbachia* core, a representative *w*Tpre gene for each of the core COGs was queried against a database of the protein coding sequences of *Rickettsia rickettsii*, *Ehrlichia chaffeensi*s, and *A. marginale*, (respective GenBank Accession Numbers CP003318, CP000236, and CP001079) using BLASTP. A cutoff e-value of 1e-10 was used to determine significance. The comparison of the core was done with both the 496-COG core (excluding *w*Wb and *w*Gmm) and the 436-COG core (excluding only *w*Wb, and *w*Gmm included).

### Analysis of genome content and ankyrin genes

Role category annotations from the IGS annotation pipeline were used to compare genome content across 17 *Wolbachia* strains, excluding unannotated genes. The number of genes in each role category for each genome was plotted according to standard deviation, then subjected to a Principle Components Analysis (PCA) based on the standardized proportion of genes in each role category, using prcomp in R version 3.1.2 (R Core Team 2014). Due to the high variance of the hypervariable “mobile and extrachromosomal element functions” category, a second PCA analysis was performed after removing the category and recalculating proportions.

The term “ankyrin” was queried against all gene annotations, and the number of positive matches was tabulated for each genome. The number of ankyrin repeat-containing genes was plotted in R, and a Mann–Whitney *U*-test was used to test for a significant difference in abundance between supergroups A and B. Supergroups C, D, and F were not included in the statistical analyses due to the small number of sequenced genomes available for those groups.

### Identification of truncated ORFs in wTpre

The nucleotide sequence of all *w*Tpre genes determined not to be a member of any orthologous clusters (see *Results*) were queried against a database of all *Wolbachia* genes from the remaining 16 genomes using BLASTN. The full nucleotide sequence of the best match was then queried back against the *w*Tpre genome sequence to look for regions of homology beyond the *w*Tpre gene ORF. To be further considered as evidence of protein sequence truncation, the BLASTN best match to the genome was required to meet an 85% identity cutoff, and the best match had to align to *w*Tpre across at least 70% of its length, or at least three times the length of the *w*Tpre gene in question. Alignments that passed these quality measures were scanned for the presence of mutations that would result in premature stop codons, and categorized by mutation type. ORF length comparisons were performed in R and a Mann–Whitney *U*-test was used to determine significance.

### Comparison to inactive genes in Wolbachia strain wAu

The set of *w*Mel genes that were found to be potentially inactive in *Wolbachia* strain *w*Au (Sutton *et al.* 2014) was compared to the *w*Tpre gene set. *w*Au was not included in previous analyses because it was published after COG assessment was completed. The *w*Mel genes were classified as either: 1) having an ortholog in *w*Tpre (as determined by Sybil COG assessment), 2) being truncated in *w*Tpre (as determined by the homolog of a truncated *w*Tpre gene sharing COG membership with the respective *w*Mel gene), or 3) absent in *w*Tpre.

### Data availability

The *T. pretiosum* colony used for sequencing is available upon request. Supplemental Material, Table S1 contains a detailed breakdown of the counts of genes in each role category and subcategory, for each *Wolbachia* strain, as annotated by IGS. Table S2 provides complete BUSCO results for all *Wolbachia* strains. Table S3 is the *w*Tpre “unique genes” considered in truncation analyses. Table S4 contains comparisons of truncated genes in *w*Au and *w*Tpre. The *w*Tpre Whole Genome Shotgun project has been deposited at DDBJ/EMBL/GenBank under the accession LKEQ00000000. The version described in this paper is version LKEQ01000000.1.

## Results

### The wTpre genome: a parthenogenesis-inducing Wolbachia strain

The genome sequence of *w*Tpre was extracted from a whole genome assembly of its host, *T. pretiosum*, performed as a part of the i5k genome project (A. R. I. Lindsey *et al.*, unpublished results). The *w*Tpre genome was recovered in a single scaffold, composed of nine contigs. The scaffold was 1,133,709 bp in length, and BLASTN searches against the NCBI GenBank database revealed 97% nucleotide similarity to the *Wolbachia* symbiont *w*Pip_Pel infecting *Culex quinquefasciatus* (GenBank accession number: AM999887). No other bacterial sequence was identified in the *T. pretiosum* assembly. Average scaffold coverage for the *Wolbachia* scaffold was the lowest of all scaffolds in the i5k genome project assembly, indicating that the recovered genome is not the result of a lateral transfer into the *T. pretiosum* genome (*Wolbachia* scaffold = 35.6 × coverage, *T. pretiosum* assembly = 232.7 × coverage). The *w*Tpre genome was structurally and functionally annotated with the Institute for Genome Sciences (IGS) pipeline at the University of Maryland (http://ae.igs.umaryland.edu/cgi/index.cgi, Galens *et al.* 2011), revealing 1405 ORFs, 35 tRNA coding genes, and a single set of rRNA genes (one each of 5S, 23S and 16S), giving a coding density of 81.8%. The size and number of coding sequences fell within the range of previously sequenced *Wolbachia* genomes (Table 1). While the arthropod-infecting *Wolbachia* genomes are known to carry a large number of mobile elements, the *w*Tpre genome was depauperate in these features. Only nine genes related to prophage function, and 14 transposon function genes were identified in the genome (Table S1).

### Genome completeness and phylogenetic relationships

Seventeen previously published *Wolbachia* genomes, representing supergroups A–D and F, were examined alongside the *w*Tpre genome in phylogenetic and comparative analyses (Table 1). All genomes were reannotated with the same IGS pipeline used to annotate *w*Tpre. BUSCO (Simão *et al.* 2015) was used to scan for the 40 core bacterial genes defined by Mende *et al.* (2013) to estimate completeness for each sequenced genome based on the proportion of missing BUSCO genes. Scores from these analyses are reported in Table 1. Notably, none of the *Wolbachia* strains, including completely sequenced genomes, contained all 40 BUSCO genes. All 18 strains are missing the BUSCO orthologs that encode for ribosomal proteins S7, L11, L4, and L14 (COG0049, COG0080, COG0088, and COG0093, respectively). The *w*Wb strain (from the nematode *Wuchereria bancrofti*) appeared to be an outlier, as 22 of the 40 orthologs were missing or fragmented (Table S2). Additionally, *w*Wb was missing a duplication of COG0552 (Signal recognition particle GTPase) that is present in all 17 other strains. The draft *Wolbachia* genomes have BUSCO scores that fall within the range of scores from the complete genomes, with the exception of *w*Wb. The *w*Wb assembly is the expected size for a *Wolbachia* genome, but has an abnormally large number of ORFs (n = 2144), almost 600 more than the other *Wolbachia* genomes (Table 1). For these reasons, the *w*Wb strain was excluded from additional analyses.

**Figure 1:**
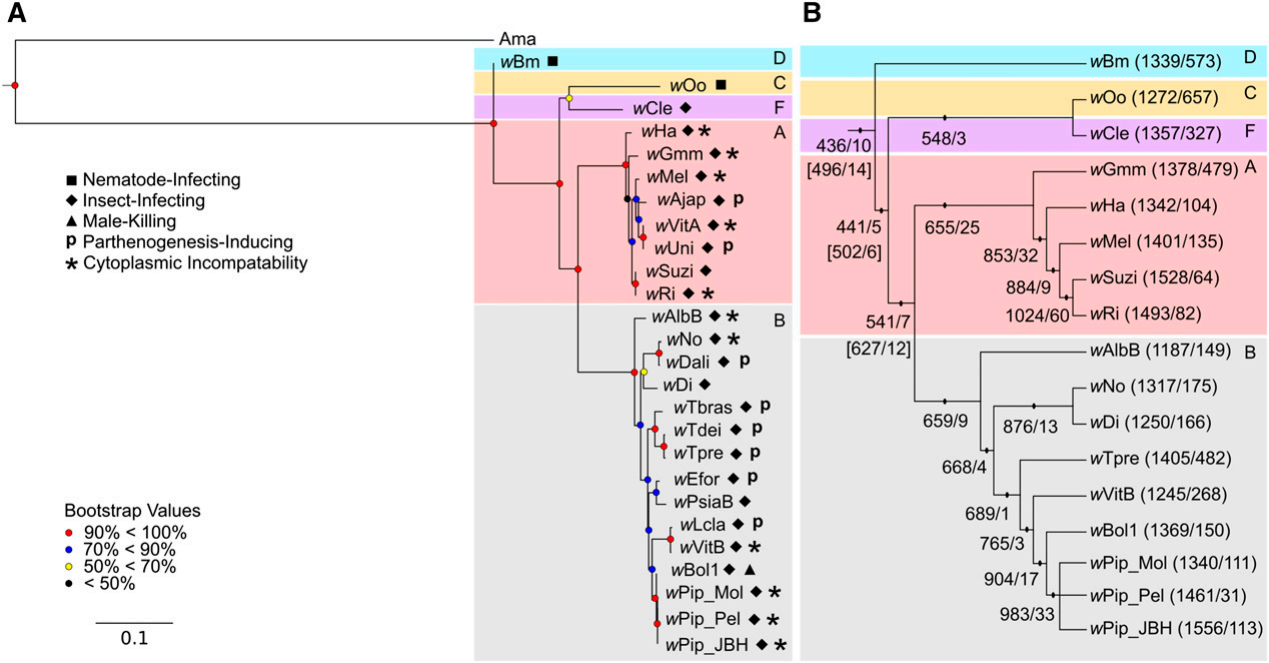
Phylogenetic relationships of *Wolbachia*. (A) Phylogeny inferred with RAxML from a nucleotide supermatrix of the five *Wolbachia* MLST (multi locus sequence typing) genes using 1000 bootstrap replicates. Supergroups are shown in colored boxes, and labeled in the top right corner of each box. Symbols next to taxa denote *Wolbachia* host and phenotypes. Colors at nodes indicate bootstrap values. *Anaplasma marginale* str. *Florida* “Ama” is the outgroup. (B) Cladogram of *Wolbachia* inferred with RAxML using the same methods as in Figure 1A, but analyzing only the strains with sequenced genomes. Numbers in parentheses next to taxon names represent, on the left, the number of genes in the genome, and on the right, the number of genes unique to that genome. Numbers corresponding to points on internodes represent, on the left, the number of core cluster of orthologous genes (COGs) for that clade, and on the right, the number of COGs unique to that clade. Numbers in square brackets represent alternative core and unique genome sizes for the respective clade, calculated without *w*Gmm. Colored boxes denote supergroups, with labels in the top right corner.

Phylogenetic reconstruction based on maximum likelihood analysis was conducted using Multilocus Sequence Typing (MLST) genes (Baldo *et al.* 2006) to determine relationships among the PI-*Wolbachia*. This analysis confirms multiple independent origins of PI-*Wolbachia*, placement of the *w*Tpre strain in the B supergroup, and the monophyly of the *Trichogramma*-infecting *Wolbachia* (Figure 1A). All supergroups with multiple members were recovered as monophyletic. The major arthropod-infecting lineages, supergroups A and B, formed a monophyletic clade, and supergroups C and F also formed a monophyletic clade. The nematode-infecting supergroup D was sister to the rest of the *Wolbachia* lineage. The *w*Pip strains have identical MLST sequences, and are represented as a polytomy.

### The core Wolbachia genome

The core genome of the 17 *Wolbachia* strains was made up of 436 COGs (Figure 1B). The core genomes of the A (655 COGs) and B (659 COGs) supergroups were similar in size despite the B supergroup being represented by four more strains than the A supergroup. Together, these two supergroups had a core genome of 541 COGs. As expected, the inclusion of additional supergroups led to a reduction in the size of the core genome. Sampling more heavily among more distantly related groups yielded a decrease in shared similarities. It is important to note that the positions of *w*Gmm and *w*Ha have changed: in the phylogenetic reconstruction including more strains (Figure 1A), *w*Ha is sister to the rest of the A supergroup and *w*Gmm is sister to the rest of the A supergroup when the phylogeny is reconstructed with only the strains for which genomes are available (Figure 1B). That node in both trees is supported by a bootstrap value of 100, so we kept the topologies and calculated core and unique genome sizes with *w*Gmm as sister to the rest of the A supergroup.

The size of the core genome for the eight *Wolbachia* strains with completely sequenced genomes (*w*Bm, *w*Cle, *w*Mel, *w*No, *w*Oo, *w*Pip_Pel, *w*Pip_Mol, and *w*Ri) was 511 COGs. Inclusion of *w*Ha, which has a genome assembly of a single scaffold with two gaps, did not reduce the core size. Addition of *w*Tpre, the remaining single-scaffold assembly, only reduced the core genome by one COG, to 510 COGs, indicating that the *w*Tpre assembly is relatively complete. These 10 complete and single-scaffold genomes were used to determine which genome(s) were having the largest effect on the final core genome size of all 17 strains. One at a time, the core genome was determined for the aforementioned 10 genomes, plus one of the seven remaining assemblies. *w*Di and *w*Suzi had a small effect on the core size, each resulting in one less COG in the core. *w*Pip_JBH reduced the core genome by two COGs. *w*AlbB and *w*Bol1 were each responsible for a loss of three COGs from the core, and *w*VitB for five COGs. The *w*Gmm strain had the most drastic effect on the size of the *Wolbachia* core, as the *w*Gmm assembly (infecting the tsetse fly *Glossina morsitans morsitans*) is missing 63 of the 510 COGs found in the 10 complete and single-scaffold genomes. Its low BUSCO score (Table 1), in combination with the effect on the core genome, indicate that a significant portion of sequence data may be missing or misassembled for *w*Gmm. Elimination of *w*Gmm from the analysis resulted in a core *Wolbachia* genome of 496 COGs for the remaining 16 strains, which is likely closer to the true size of the *Wolbachia* core. This 496 COG core was searched against *R. rickettsii*, *E. chaffeensi*s, and *A. marginale*. Fourteen *Wolbachia* core COGs did not have hits to the other Rickettsiales: 11 hypothetical or predicted proteins, a cutA1 divalent ion tolerance family protein, a surface antigen family protein, and a nitroreductase family protein. Four of these 14 *Wolbachia*-unique COGs, all conserved hypothetical proteins, are missing from the 436-COG core that includes *w*Gmm.

### Ordination of Wolbachia strains based on genome content

**Figure 2:**
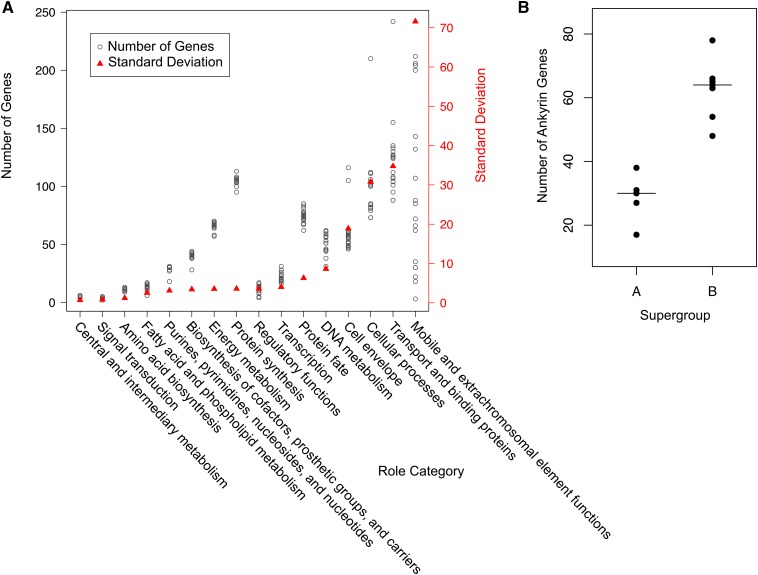
Gene content of *Wolbachia*. (A) The numbers of genes in each role category, for each *Wolbachia* genome are plotted with open circles and correspond to the left axis. Role categories are sorted by standard deviation, represented by the red triangles, and the right axis. (B) Number of ankyrin repeat-containing genes per genome, by supergroup. The B supergroup has a significantly higher number of ankyrin genes than the A supergroup (Mann–Whitney *U*-test, *P* = 0.003).

**Figure 3:**
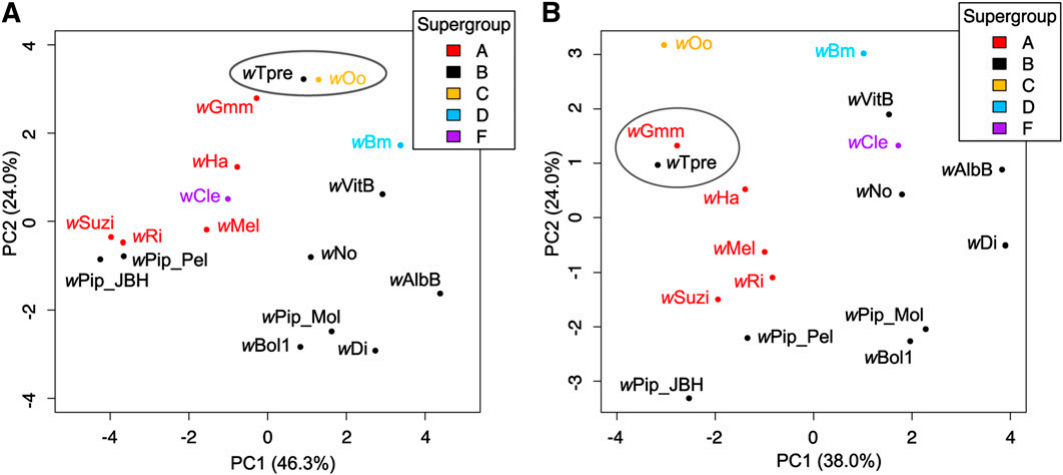
Principal components (PC) analysis of *Wolbachia* genomes based on proportion of annotated genes devoted to each role category, with *w*Tpre and closest neighbor circled. (A) All annotated role categories analyzed. The strongest factor loadings along PC1 (46.3% of total variance) and PC2 (34.0% of total variance), respectively, are energy metabolism and regulatory functions. (B) Mobile and extrachromosomal elements functions category excluded. The strongest factor loadings along PC1 (38.0% of total variance) and PC2 (24.0% of total variance), respectively, are cellular processes and DNA metabolism.

The number of genes in each role category, for each genome, as determined by the IGS annotation pipeline, was used in comparative analyses of genome content. The role categories with the most variation in gene number per genome were: mobile and extrachromosomal element functions, transport and binding proteins, and cell envelope (Figure 2A). *Wolbachia* genomes showed little variance in the number of genes devoted to central intermediary metabolism, signal transduction, and amino acid biosynthesis. All *Wolbachia* genomes had a high (median = 106), but relatively conserved number of genes devoted to protein synthesis. Principal Components Analysis (PCA) was used to visualize the similarity of genomes based on the proportion of genes in each of these role categories (Figure 3A). While the A supergroup genomes ordinate to the upper left quadrant, the B supergroup strains showed greater diversity in genome content across strains. Bed bug-infecting *w*Cle clustered with the distantly related, yet also arthropod-infecting, A supergroup strains, although phylogenetically *w*Cle belongs to the F supergroup (Rasgon and Scott 2004). *w*Tpre’s closest neighbor in the genome content-based ordination was the obligate, nematode-infecting *w*Oo strain. We suspect that the highly variable number of genes in the mobile and extrachromosomal element functions role category could strongly influence these patterns. Therefore, proportions were recalculated without this category and again subjected to PCA (Figure 3B). Without the mobile and extrachromosomal element functions role category, the *w*Cle genome neighbored B supergroup strains, and the *w*Tpre genome neighbored the group of A supergroup strains. This category had a dominant effect on the ordination of *w*Tpre and *w*Cle. However, the overall pattern of a loose A supergroup cluster and B supergroup diversity was maintained in the absence of the mobile and extrachromosomal element functions category, indicating support from other role categories for this patterning.

### Supergroup B has significantly more ankyrin repeat-containing genes

We specifically looked at the number of ankyrin repeat-containing (ANK) genes in each of the *Wolbachia* genomes. ANK genes are involved in protein-protein interactions and are rare in bacteria, but are found in *Wolbachia*, where they may modulate host phenotypes (Iturbe-Ormaetxe *et al.* 2005; Papafotiou *et al.* 2011). The *w*Tpre strain has 54 ANK genes. With 48 ANK genes, the *w*AlbB strain has the fewest number of ANK genes in the B supergroup. We demonstrate a significant difference in the number of ANK genes between supergroups A and B (Mann Whitney-U, *P* = 0.003) (Figure 2B). The B supergroup has, on average, more than double the number of ANK genes than any other supergroup. The median number of ANK genes in supergroup A is 30, and in supergroup B is 64. While supergroups C, D, and F were not subjected to statistical analysis due to the low number of representative genomes available, the numbers of ANK genes present in those genomes was low when compared to supergroup B. The *w*Oo (C), *w*Bm (D), and *w*Cle (F) genomes have 3, 20, and 39 ANK genes, respectively.

### “Unique” wTpre genes are derived from truncated versions of Wolbachia genes

**Figure 4:**
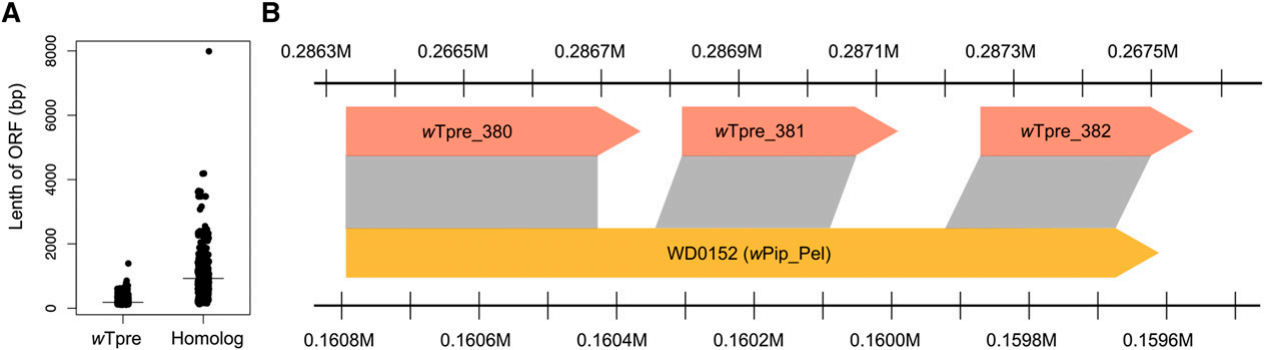
Evidence for truncation in *w*Tpre genes. (A) Length of *w*Tpre “unique genes” and their homologous genes from other *Wolbachia* genomes. There is a significant difference in the size of the *w*Tpre unique gene set as compared to their homologous counterparts (Mann–Whitney *U*-test, *P* < 0.0001). (B) Schematic representation of *w*Tpre coding frame truncation and fragmentation. The *w*Tpre “unique genes,” *w*Tpre_380, *w*Tpre_381, and *w*Tpre_382, are homologous to sequential locations in the WD0152 gene from *w*Pip_Pel. A frameshift mutation at base pair 421 in *w*Tpre_380 resulted in a premature stop codon and the subsequent annotation of downstream ORFs (open reading frames), or “postframeshift” ORFs.

The newly sequenced *w*Tpre strain has one of the largest sets of “unique genes,” and the largest of all the arthropod-infecting *Wolbachia* strains, with 482 genes not assigned any orthologs (Figure 1B). This represents 34% of the total genes in the *w*Tpre genome. Nucleotide BLAST searches of the *w*Tpre “unique genes” against a database of all the other coding sequences from the other *Wolbachia* genomes in Table 1 reveal that 367 of *w*Tpre “unique genes” show similarity with other *Wolbachia* genes (Table 2). However, the predicted coding regions of *w*Tpre “unique genes” were on average 77.5% shorter than their corresponding homologs in other *Wolbachia* genomes (Mann–Whitney *U*-test, *P* < 0.0001) (Figure 4A). The significant difference in size could indicate that these genes are truncated versions of the coding sequence, either due to deletions, or premature stop codons. To explore this, the nucleotide sequences of the best matches were aligned to the *w*Tpre genome sequence to look for homology of the *w*Tpre “unique gene” up- and downstream of the ORF. Of the 367 *w*Tpre “unique genes” with sequence similarity to other *Wolbachia* genes, 86 genes were excluded from analyses based on low identity values and/or lack of evidence for up/downstream homology, and 281 genes showed evidence of truncation of the predicted protein sequence and potential pseudogenization due to nonsense and frameshift mutations (Table 2 and Table S3). Many of the *w*Tpre “unique genes” occur in tandem, where an early frameshift or nonsense mutation resulted in a premature stop codon, and subsequent annotation of additional short, downstream ORFs with sequence homology to the downstream portions of the same ORF in the other *Wolbachia* genome. Figure 4B shows a schematic representation of this phenomenon, where the *w*Tpre “unique genes” *w*Tpre_380, *w*Tpre_381, and *w*Tpre_382 all align to sequential portions of the *w*Pip_Pel gene, WD0152. A single base pair deletion at position 421 in *w*Tpre_380, relative to *w*Pip_167, resulted in a premature stop codon. The intergenic spaces between these *w*Tpre “unique genes” also showed sequence similarity to corresponding locations in the *w*Pip_Pel gene. The short ORFs downstream of the nonsense or frameshift mutation are hereafter referred to as “postnonsense” or “postframeshift” ORFs, respectively. In the *w*Tpre genome, 52% (n = 146) of these “unique genes” with evidence of truncation were postframeshift ORFs (Table 2 and Table S3). The coding frame truncated *w*Tpre genes were more likely to have a hypothetical annotation than their counterparts from other *Wolbachia* genomes (Chi-Square, *P* < 0.0001). Of the 281 truncated *w*Tpre genes, 149 (53%) had a hypothetical annotation. This contrasts to the 188 genes that the truncated *w*Tpre genes match to, where only 62 (33%) had a hypothetical annotation. Of the truncated *w*Tpre genes, 57 are of phage or transposon origin, and 45 are homologs of ANK genes. Therefore, we conclude that the majority of these “unique genes” are artifacts of ORF prediction, and are actually degenerated protein coding sequences of genes found in other *Wolbachia*.

**Table 2:** Classification of *w*Tpre “unique genes”

“Unique Genes” with Evidence of Truncation		“Unique Genes” Without Evidence of Truncation	
Nonsense mutation	26	No match to other *Wolbachia* genes	115
Postnonsense	76	Low identity score of alignment	7
Frameshift mutation	30	Homolog is shorter than *w*Tpre gene	11
Postframeshift	139	No up/downstream homology	68
Poststart codon mutation	10		
Total truncations	281	Total excluded	201

### Comparison to inactive genes in Wolbachia strain wAu

The genome for the *w*Au strain infecting *Drosophila simulans* was recently sequenced, and also found to be missing or have potentially inactive versions of homologous genes present in the closely related *w*Mel strain (Sutton *et al.* 2014). While *w*Mel induces strong CI, *w*Au has lost this function (Hoffmann *et al.* 1996). All of the 46 *w*Mel genes found to be inactive in *w*Au were members of COGs, and were not unique to *w*Mel. Of these 46 *w*Mel genes, 36 were either absent (n = 24), truncated (n = 9), or “unique genes” that did not meet criteria to be considered truncations (n = 3) in the *w*Tpre genome (Table S4). Ten of the *w*Mel genes shared the same fate in both the *w*Tpre and *w*Au genomes. Five hypothetical proteins, an ANK protein, and DNA repair protein RadC, are absent in both *w*Tpre and *w*Au. Multidrug resistance protein D and a hypothetical protein both have frameshift mutations in *w*Tpre and *w*Au. Lastly, a prophage gene has a nonsense mutation in both strains.

## Discussion

The *w*Tpre assembly represents the most complete genome sequence of a parthenogenesis-inducing *Wolbachia* to date. This particular PI-*Wolbachia* strain is required for reproduction in its host; attempts to initiate *Wolbachia*-free replicates of this *Trichogramma* colony, following protocols from Stouthamer *et al.* (1990), have not been successful (*e.g.*, Russell and Stouthamer 2011). The only other available PI-*Wolbachia* genome is strain *w*Uni from the parasitic wasp *Muscidifurax uniraptor*, an A supergroup *Wolbachia* (Klasson *et al.* 2009). *w*Uni was not included in analyses as the record contains only partial genome data that was generated by amplification with primers based on the *w*Mel genome.

In some ways, the *w*Tpre genome is similar to the other arthropod-infecting strains. *w*Tpre contains a large number of ANK genes, as is common in the *Wolbachia* clade. With regards to the number of phage genes, the *w*Tpre genome is more similar to the obligate, nematode-infecting *Wolbachia*: *w*Tpre contains nine annotated phage genes and 14 transposon function genes. As a comparison, the same annotation pipeline identified 55 prophage function genes and 132 transposon function genes in the *w*Pip_Pel genome, and 30 prophage and 81 transposon genes in the *w*Mel strain (infecting *Drosophila melanogaster*). This corroborates previous analyses that discovered a diversity of phages in many other arthropod-infecting *Wolbachia*, but no evidence of functional bacteriophages in the *Trichogramma*-infecting *Wolbachia* (Gavotte *et al.* 2007). Phylogenetic analyses confirmed the multiple origins of PI-*Wolbachia*, and monophyly of the *Trichogramma*-infecting strains (van Meer *et al.* 1999). The relationship of the supergroups using the five MLST genes (Baldo *et al.* 2006) replicated results from phylogenomic analyses using 90 informative loci (Gerth *et al.* 2014).

We attempted to assess the completeness of the *Wolbachia* genomes using the BUSCO pipeline and 40 core bacterial genes. Completely sequenced genomes varied widely in the number of genes recovered, indicating that this gene set may not be ideal for assessing completeness in *Wolbachia*. Four ribosomal proteins were absent from all *Wolbachia* genomes. Genome sequencing of the primary-symbionts of insects has revealed that not all ribosomal proteins are retained in these highly reduced genomes (McCutcheon 2010). While *Wolbachia* is not considered a primary-symbiont, and is not strictly maternally transmitted (Raychoudhury *et al.* 2009), some degree of genome reduction has taken place. There was a trend toward lower BUSCO scores in the obligate *Wolbachia* strains, indicating more extensive reductions in genomic content.

Due to the draft status of some of the *Wolbachia* genomes, we relied on the proportions of genes in role categories to assess similarity of genome content. The *w*Tpre strain clusters with the nematode infecting strains when mobile and extrachromosomal elements are included, likely driven by the similarity in the number of phage genes. Without this category of genes, *w*Tpre neighbors A supergroup *Wolbachia*. The ordination of *w*Cle also changes drastically when the mobile and extrachromosomal element genes are removed from the analysis, going from neighboring A supergroup strains to neighboring B supergroup strains. While the mobile and extrachromosomal elements role category appears to have a dominant effect on ordination for certain strains, the overall pattern of the A and B supergroups was more strongly supported.

The size of the core genome here (496 COGs) was lower than estimates from previous studies. Duplouy *et al.* (2013) estimated a core of 654 genes based on five strains (from three supergroups): *w*Bol1, *w*Pip_Pel, *w*Mel, *w*Ri, and *w*Bm. Similarly, Ishmael *et al.* (2009) used exponential regression to estimate a core genome size of 621 genes, but their study examined only *Drosophila*-infecting *Wolbachia* strains. It is likely that our inclusion of additional *Wolbachia* strains, from more diverse hosts and supergroups, is responsible for the smaller core genome size. Comparison of the core *Wolbachia* genome to other members of the Rickettsiales revealed that only 2.8% of the core is unique to *Wolbachia*. This finding parallels the discovery of high conservation of two-component systems across 12 *Wolbachia* strains, *A. phagocytophilum*, and *E. chaffeensis* (Christensen and Serbus 2015). These similarities with other closely related rickettsial pathogens may indicate that the core genome comprises genes required for life within an arthropod host, and that the accessory genomes are responsible for the phenotypes that various strains induce.

In *w*Tpre, 482 (34%) of the ORFs were apparently unique: the largest number of any of the arthropod-infecting strains. Only the two nematode-infecting strains, *w*Bm and *w*Oo, had more “unique genes” than *w*Tpre. This may be a feature of the obligate nature of the symbiotic relationships that these strains share with their hosts. However, *w*Bm and *w*Oo are the only representatives from their respective supergroups, and it is likely that inclusion of additional C and D supergroup members would result in a reduction in the number of “unique genes” found in these strains. The *w*Gmm strain also contained a high number of “unique genes”. This may be a result of a problematic assembly, as *w*Gmm had one of the lower BUSCO scores and was responsible for a drastic effect on the size of the core *Wolbachia* genome.

Examination of the *w*Tpre “unique genes” showed evidence for coding frame truncation in 281 genes, representing 20% of the ORFs in the genome. This is likely an underestimate of the amount of truncation in *w*Tpre. Stringent filtering of sequence similarity, and of up- and downstream homology, did not allow for identifying truncation in rapidly evolving genes, or genes that may have been truncated or fragmented through genomic rearrangements or deletions. Mutations resulting in downstream postnonsense and postframeshift ORFs were not exclusively located in genes identified as unique to *w*Tpre. If the mutation occurred too early in the coding sequence, the ORF was too short to be considered a gene by the IGS pipeline. Conversely, mutations that occurred more 3′ in the coding sequence left an ORF long enough to be considered orthologous with other *Wolbachia* genes, but could still result in the annotation of short downstream *w*Tpre “unique” ORFs. In *w*Tpre, truncated genes were more likely to carry a hypothetical annotation, despite the fact that homologs from other *Wolbachia* genomes were often assigned a function. One explanation for this may be the frameshift mutations that result in a change of amino acid sequence, and the loss of recognized functional domains or motifs that would assist in assigning function to the gene. Additionally, the fragmentation of a gene into several ORFs would lead to a functional domain or motif only being associated with one of the resulting ORFs, thus making functional assignments difficult for the other ORFs. Therefore, we conclude that the majority of “unique genes” in *w*Tpre are actually truncated orthologs of known *Wolbachia* genes from other strains, and likely are not active protein coding genes, but artifacts of ORF prediction machinery.

A relatively small number of inactive or truncated genes were identified in *w*Au, a *Wolbachia* strain infecting *D. simulans* that does not induce strong CI, but does provide viral protection to its host. While the *w*Tpre genome contains a larger number of truncated genes, 78% of the inactive *w*Au genes were also missing or truncated in *w*Tpre, providing an overlapping set of 36 genes. Both *w*Au and presumably *w*Tpre have lost the capacity for CI induction. This overlap may indicate an important feature of the transition away from a strong CI phenotype. However, many of these genes have hypothetical gene annotations, and therefore we cannot comment on their potential functions.

We identified a significantly higher number of ANK genes in the B supergroup *Wolbachia* strains. ANK genes are unusual in bacteria, and it has been hypothesized that phages, transposons, and recombination may have played a role in proliferation of the ANK gene repertoire in *Wolbachia* (Siozios *et al.* 2013b; Iturbe-Ormaetxe *et al.* 2005). The *w*Tpre strain has 54 ANK genes, despite not having associated bacteriophages and having a reduced number of mobile elements. *w*Tpre may have lost its mobile elements and bacteriophages more recently. Indeed, 57 of the 281 truncated *w*Tpre genes (20.2%) are versions of *Wolbachia* genes with phage or transposon function.

We hypothesize that the extensive protein coding frame truncations present in *w*Tpre reflect the change in reproductive phenotype from CI to PI. In *Trichogramma*, fixation of asexual reproduction can occur through changes in the host genome, which makes *Wolbachia* essential to the production of female offspring; so called virginity mutations (Russell and Stouthamer 2011; Stouthamer *et al.* 2010). While this *w*Tpre strain does infect a host that is dependent upon *w*Tpre’s parthenogenesis-induction, not all *Trichogramma*, or even all *T. pretiosum*, have this dependent relationship with their resident *Wolbachia* strains. Sequencing of additional *Trichogramma*-infecting *Wolbachia* strains is necessary to determine whether or not these coding frame truncations are pervasive across all PI-*Wolbachia*, just the *Trichogramma*-infecting *Wolbachia*, or are unique to strains such as *w*Tpre that infect irreversibly asexual hosts.

## Supplementary Material

Supplemental Material
