# Supplementary material for: Comparative Genomics of a Parthenogenesis-Inducing Wolbachia Symbiont
Source: G3 (Bethesda). 2016 May 16;6(7):2113–23. doi: 10.1534/g3.116.028449 (PMC4938664; doi:10.1534/g3.116.028449)
Supplement: Supplemental Material [file supp_6_7_2113__index.html]

Comparative Genomics of a Parthenogenesis-Inducing Wolbachia Symbiont — Supplemental Material 

# Comparative Genomics of a Parthenogenesis-Inducing *Wolbachia* Symbiont

## Supplemental Material for Lindsey *et al.*, 2016

**Files in this Data Supplement:**

- Table S1 - Table S1 contains a detailed breakdown of the counts of genes in each role category and sub-category, for each *Wolbachia* strain, as annotated by IGS. (.xls, 53 KB)
- Table S2 - Table S2 provides complete BUSCO results for all *Wolbachia* strains. (.xls, 45 KB)
- Table S3 - Table S3 is the *w*Tpre "unique genes" considered in truncation analyses. (.xls, 85 KB)
- Table S4 - Table S4 contains comparisons of truncated genes in *w*Au and *w*Tpre. (.xls, 31 KB)
